# Investigating the impact of cartilaginous endplate herniation on recovery from percutaneous endoscopic lumbar discectomy

**DOI:** 10.1186/s13018-024-04746-4

**Published:** 2024-04-25

**Authors:** Zenghui Zhao, Hao Qi, Chenchen Wang, Anqi Zhao, Feiyu Zu, Jianzhou Zhang, Mengzi He, Hongru Yuan, Ao Yang, Chenxi Wang, Di Zhang

**Affiliations:** 1 Department of Spine Surgery, The Third Hospital of Hebei Medical University, Shijiazhuang, 050051 China; 2 Hebei Medical University School of Basic Medical Sciences, Shijiazhuang, China

**Keywords:** Percutaneous endoscopic lumbar discectomy (PELD), Lumbar disc herniation (LDH), Herniation of cartilaginous endplates, Postoperative pain, Modic changes

## Abstract

**Objective:**

This study aimed to evaluate the influence of herniation of cartilaginous endplates on postoperative pain and functional recovery in patients undergoing percutaneous endoscopic lumbar discectomy (PELD) for lumbar disc herniation (LDH).

**Methods:**

A retrospective analysis was conducted on 126 patients with LDH treated with PELD at the Third Hospital of Hebei Medical University from January 2021 to January 2022. Whether cartilaginous endplates had herniated was identified by analyzing these specific findings from MRI scans: posterior marginal nodes, posterior osteophytes, mid endplate irregularities, heterogeneous low signal intensity of extruded material, and Modic changes in posterior corners and mid endplates. Patients were assessed for postoperative pain using the Visual Analogue Scale (VAS) and functional recovery using the Oswestry Disability Index (ODI) and Modified MacNab criteria. Statistical analyses compared outcomes based on the presence of herniation of cartilaginous endplates.

**Results:**

Patients with herniation of cartilaginous endplates experienced higher pain scores early postoperatively but showed significant improvement in pain and functional status over the long term. The back pain VAS scores showed significant differences between the groups with and without herniation of cartilaginous endplates on postoperative day 1 and 1 month (*P* < 0.05). Leg pain VAS scores showed significant differences on postoperative day 1 (*P* < 0.05). Modic changes were significantly associated with variations in postoperative recovery, highlighting their importance in predicting patient outcomes. In patients with herniation of cartilaginous endplates, there were statistically significant differences in the back pain VAS scores at 1 month postoperatively and the ODI functional scores on postoperative day 1 between the groups with and without Modic changes (*P* < 0.05). There were no significant differences in the surgical outcomes between patients with and without these conditions regarding the Modified MacNab criteria (*P* > 0.05).

**Conclusion:**

Herniation of cartilaginous endplates significantly affect early postoperative pain and functional recovery in LDH patients undergoing PELD. These findings emphasize the need for clinical consideration of these imaging features in the preoperative planning and postoperative management to enhance patient outcomes and satisfaction.

## Introduction

Lumbar Disc Herniation (LDH) is one of the common causes of lower back pain and sciatica [1]. With the development of minimally invasive surgical techniques, Percutaneous Endoscopic Lumbar Discectomy (PELD) has become an important method for treating LDH. PELD is widely applied and recognized over the past few decades due to its advantages such as minimal trauma, quick recovery, short hospital stay, and clinical outcomes comparable to open surgery [2].

**Fig. 1:**
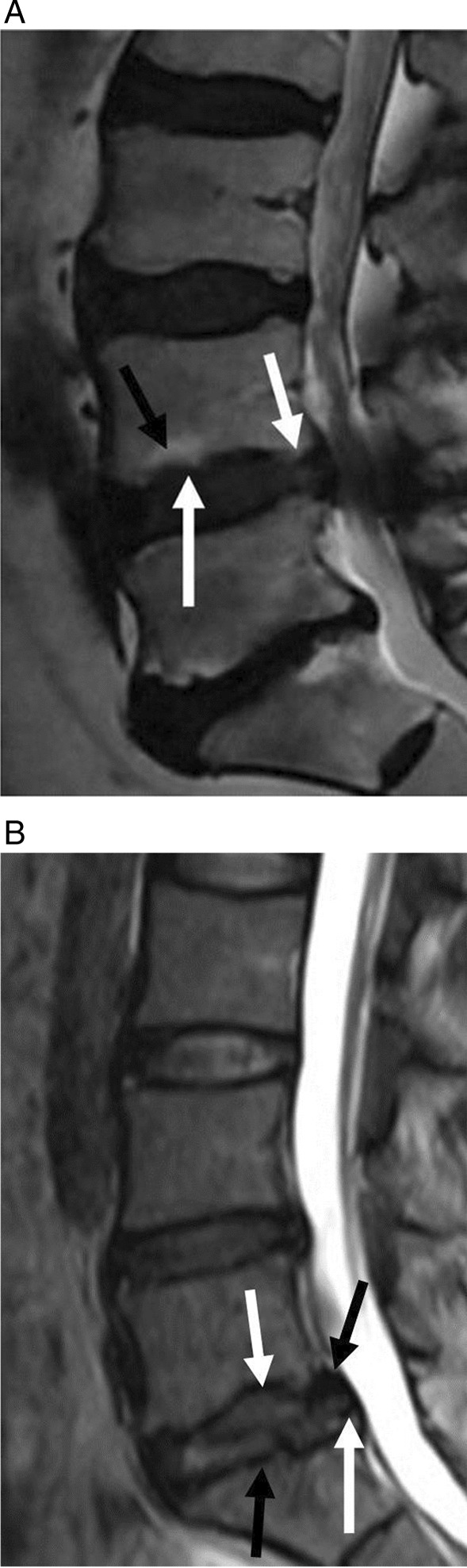
**A** The black arrow above indicates Modic changes of the mid endplates, the white arrow above indicates Modic changes of the posterior corners of the endplates, and the white arrow below indicates irregularities of the mid endplates. **B** The white arrow above indicates posterior marginal nodes, and the black arrow above indicates dorsal corner defects. The white arrow below indicates heterogeneous low signal intensity of extruded material. The black arrow below indicates mid endplates irregularities

Despite the significant clinical success of PELD, the surgical outcomes are not always ideal. Postoperative pain management and functional recovery are key factors affecting patient satisfaction and quality of life [3]. Studies have shown that herniation of cartilaginous endplates and Modic changes [4] are important predictors of postoperative pain and functional recovery in LDH patients. The occurrence of herniation of cartilaginous endplates and Modic changes not only reflects the degree of degenerative changes in the intervertebral disc but is also closely related to increased early postoperative pain scores and differences in functional recovery [4]. Among these, there is extensive research on Modic changes, but less is known about other injuries to the endplate, such as herniation of cartilaginous endplates (Fig. 1).

In the treatment of LDH, choosing the appropriate surgical method is crucial for improving the success rate of surgery and patient satisfaction [5]. Currently, the advantages of PELD over traditional open microdiscectomy (Microdiscectomy) in terms of pain control and functional recovery have not been fully proven. Although there is a large body of literature on the safety and effectiveness of PELD [6], there is relatively less research on the impact of herniation of cartilaginous endplates and Modic changes on postoperative pain and recovery.

This study aims to systematically evaluate postoperative pain and functional recovery in LDH patients undergoing PELD treatment, with a special focus on the impact of herniation of cartilaginous endplates and Modic changes on postoperative recovery. Through this study, we hope to provide a more precise clinical decision-making basis for personalized treatment of LDH patients, further optimize LDH treatment schemes, and improve postoperative satisfaction and quality of life for patients.

## Methods

This study was conducted in accordance with the Declaration of Helsinki. Ethical approval and consent to participate in the study were obtained from the ethics committee of the Third Hospital of Hebei Medical University (Ethics Committee HB/KY-06-06/2.0) on July 25, 2022 (Approval number: K2022-067-1). All patients involved in our study provided written informed consent, and for underage patients, written informed consent was obtained from their legal guardians.

### Patients

We retrospectively collected data from patients with Lumbar Disc Herniation (LDH) who were consecutively admitted for Percutaneous Endoscopic Lumbar Discectomy (PELD) at the Third Hospital of Hebei Medical University from January 2021 to January 2022. Based on the following inclusion and exclusion criteria, 126 patients were suitable for our study. The inclusion criteria included: (1) symptoms of back pain or radicular pain; (2) imaging evidence of single-segment lumbar disc herniation associated with symptoms; (3) ineffective conservative treatment for over 3 months; (4) follow-up of at least 12 months (± 10 days). The exclusion criteria included: (1) segmental instability; (2) previous lumbar surgery; (3) cauda equina syndrome; (4) ankylosing spondylitis; (5) spinal tumor; (6) lumbar fracture; (7) tuberculosis; (8) vertebral infection; (9) rheumatic immune system diseases.

### Surgery

All surgeries were performed by an experienced spine surgeon.

All surgeries were conducted using local anesthesia. The patients were positioned on their sides, with the side to be treated facing upward. The surgeon, utilizing X-ray imagery, determined and marked the precise location for the puncture. Following this, an 18-gauge spinal needle was carefully introduced into the designated intervertebral disc space. When viewed from the side, the tip of the needle was aligned with the posterior boundary of the vertebral body. From a front-to-back perspective, this tip was positioned along the central line of the pedicle. Subsequently, a guidewire was threaded through the needle, which was then withdrawn. An initial cut was made to facilitate the insertion of a tapered, hollow obturator along the guidewire's path. This obturator was then driven into the disc space using a mallet. Following this, an oval cannula with a beveled end was introduced. An endoscopic device was then passed through this cannula, enabling the removal of the herniated disc material using specialized forceps and a radiofrequency instrument. During the decompression process, ensuring the complete decompression of the nerve root is the primary goal of the surgery. Through direct endoscopic visualization, the pathological tissues compressing the nerve root are precisely removed until the nerve root is observed to regain normal pulsation and a state of no compression. Moreover, during the surgery, precise localization and target-oriented techniques are adopted to minimize damage to the surrounding soft tissues, while maintaining the key structures of the spine to preserve its stability. Under local anesthesia, the patient remains conscious and can provide immediate feedback, allowing the surgical team to adjust the surgical strategy in real time. This interactivity ensures that the maximum protection of nerve function is achieved while fully decompressing, reaching the endpoint of the surgery,and close the surgical incision.

The patients participating in this study were all required to rest in bed within one day after surgery, and were given non-steroidal anti-inflammatory drugs and safe doses of opioid medications for pain relief according to the severity of their pain.

### Data collection

The MRI imaging assessment was conducted by senior radiologists who were blinded to the surgical outcomes, while a professional spine surgeon not involved in the surgery evaluated the patients postoperatively using tools such as the Visual Analog Scale (VAS), the Oswestry Disability Index (ODI), and the MacNab criteria.

The general data of the patient includes age, gender, height, weight, calculation of Body Mass Index (BMI), follow-up time, intervertebral disc location, and intervertebral disc level.

Using the scoring system designed by Eugene Joe et al. [7], six MRI manifestations are evaluated to diagnose disc herniation combined with cartilaginous Herniation of cartilaginous Endplates:Posterior marginal nodes, posterior osteophytes, Modic changes in posterior corners, mid endplate irregularities, Modic changes in mid endplates, and heterogeneous low signal intensity of extruded material. The AUC of this scoring system was 0.888.

Clinical outcomes are evaluated by collecting questionnaire survey results preoperatively, and 1 day, 1 month, and 1 year postoperatively. The questionnaires use the Visual Analogue Scale (VAS) for measuring the intensity of back and leg pain, and the Oswestry Disability Index (ODI) for disability. The patients' satisfaction with the clinical outcomes was evaluated using the modified MacNab criteria, which are divided into four grades: excellent, good, fair, and poor. The categories of excellent and good are considered clinically satisfactory.

Statistical analyses were performed using SPSS software (Version 27.0, Chicago, Illinois, USA). Comparisons between groups were conducted using independent sample t-tests, Chi-square tests, and Mann–Whitney U tests; comparisons within groups were conducted using paired t-tests. A P-value of less than 0.05 was considered statistically significant.

## Result

### General information

A total of 126 patients with disc herniation with and without herniation of cartilaginous endplates met the inclusion criteria. The differences in age among the preoperative demographic characteristics of the two groups of patients were statistically significant (*P* < 0.05) (Table 1), while differences in gender, BMI, etc., were not statistically significant (*P* > 0.05) (Table 1). There were no statistically significant differences in Disc level and Disc location. The difference in the Modified MacNab evaluation between the two groups of patients after surgery was not statistically significant.

**Table 1:** The general information of two groups

	With herniation of cartilaginous Endplates (n = 45)	Without herniation of cartilaginous Endplates (n = 81)	*P* value
Age (years)	50.31 ± 12.94	45.70 ± 11.41	0.042*
*Gender*			0.526
Male	24	48	0.0587
Female	21	33	
BMI (kg/m2)	21.91 ± 2.44	22.50 ± 3.10	0.248
Follow-up duration (months)	12.64 ± 0.44	12.50 ± 0.31	0.067
*Disc level*			
L3-4	2	3	0.807
L4-5	31	52	
L5-S1	12	26	
*Disc location*			
Central	11	24	0.391
Paracentral	34	57	
Modified MacNab evaluation (excellent/good/fair/poor)	29/10/4/2	47/24/7/3	0.585
Excellent/good rate	86.67%	87.65%	0.567

*P < 0.05 is considered statistically significant

### Clinical outcomes

Postoperative VAS scores and ODI were significantly reduced in both groups compared to preoperative scores (*P* < 0.05) (Table 2). There were statistically significant differences in back pain VAS scores one day and one month after surgery, and in leg pain VAS scores one day after surgery between the two groups (*P* < 0.05) (Table 2). There were no statistically significant differences in back pain VAS scores before surgery and one day after surgery, nor in leg pain VAS scores before surgery, one month after surgery, and one year after surgery (*P* > 0.05). There were no statistically significant differences in ODI scores before and after surgery between the two groups (*P* > 0.05).

**Table 2:** Clinical outcomes of with herniation of cartilaginous endplates and without herniation of cartilaginous endplates

	With herniation of cartilaginous Endplates (n = 45)	Without herniation of cartilaginous Endplates (n = 81)	*P* value
*VAS back*			
Preoperative	6.13 ± 1.18	5.89 ± 0.86	0.186
1 day after operation	2.09 ± 1.43	1.38 ± 1.02	0.002*
1 month after operation	1.40 ± 0.81	0.79 ± 0.80	< 0.001*
1 year after operation	0.51 ± 0.66	0.56 ± 0.65	0.716
*VAS leg*			
Preoperative	6.91 ± 1.47	7.01 ± 1.59	0.725
1 day after operation	2.60 ± 1.07	1.79 ± 1.23	< 0.001*
1 month after operation	1.09 ± 0.99	0.96 ± 0.95	0.486
1 year after operation	0.53 ± 0.73	0.62 ± 0.87	0.585
*ODI*			
Preoperative	63.91 ± 8.54	63.25 ± 13.53	0.766
1 day after operation	21.62 ± 13.66	22.81 ± 11.42	0.602
1 month after operation	14.58 ± 9.14	13.84 ± 6.48	0.599
1 year after operation	9.47 ± 7.56	8.78 ± 7.20	0.614

*P < 0.05 is considered statistically significant

In patients with herniation of cartilaginous Endplates, we divided them into two groups based on the presence or absence of Modic changes as the criterion (Table 3). There were statistically significant differences in the back pain VAS scores one month post-operation and the ODI scores one day post-operation between the two groups (*P* < 0.05). There were no statistically significant differences in the pre-operative, one day post-operative, and one year post-operative back pain VAS scores between the two groups (*P* > 0.05). The differences in leg pain VAS scores pre-operation and post-operation were not statistically significant in both groups. There were no statistically significant differences in the pre-operative, one month post-operative, and one year post-operative ODI scores between the two groups (*P* > 0.05).

**Table 3:** The difference in postoperative pain with or without Modic changes With herniation of cartilaginous Endplates

	With Modic changes	Without Modic changes	*P* value
*VAS back*			
Preoperative	6.07 ± 1.20	6.22 ± 1.16	0.683
1 day after operation	2.19 ± 1.54	1.94 ± 1.25	0.570
1 month after operation	1.59 ± 0.79	1.11 ± 0.75	0.048*
1 year after operation	0.52 ± 0.64	0.50 ± 0.70	0.929
*VAS leg*			
Preoperative	6.96 ± 1.37	6.83 ± 1.65	0.785
1 day after operation	2.63 ± 1.04	2.56 ± 1.14	0.827
1 month after operation	1.11 ± 1.05	1.06 ± 0.93	0.854
1 year after operation	0.56 ± 0.75	0.50 ± 0.70	0.803
*ODI*			
Preoperative	64.81 ± 9.44	62.56 ± 6.98	0.362
1 day after operation	27.48 ± 13.75	12.83 ± 7.59	< 0.001*
1 month after operation	15.33 ± 10.13	13.44 ± 7.54	0.478
1 year after operation	10.00 ± 7.74	8.67 ± 7.42	0.565

*P < 0.05 is considered statistically significant

## Discussion

Both groups of patients showed significant improvement in pain scores and functional status at follow-ups of 1 day, 1 month, and 1 year, consistent with the results of previous studies. The modified MacNab criteria indicated that patient satisfaction in both the herniation of cartilaginous endplates group and the non-herniation of cartilaginous endplates group was acceptable, demonstrating that the use of PELD to treat LDH is equally effective, regardless of the presence or absence of herniation of cartilaginous endplates.

Our research found that the average age of patients with herniation of cartilaginous endplates (n = 45) was greater than that of patients without herniation of cartilaginous endplates (n = 81), with the difference being statistically significant (*P* < 0.05). However, the relevant literature notes that it is more common in young people and during the growth period [8, 9]. In all probability, the late presentation may be the reason for an acute exacerbation on a chronic basis. The thickness of the vertebral endplate and bone mineral density (BMD) showed no significant correlation with age, suggesting that the physical properties of the endplate might not undergo significant changes with aging. However, vertebral endplates adjacent to intervertebral discs with a higher degree of degeneration exhibited greater thickness, indicating a certain correlation between disc degeneration and structural changes in the endplate [10]. During our data collection process, we also observed that patients with herniation of cartilaginous endplatess were more likely to experience disc degeneration. Numerous studies have found a significant correlation between disc degeneration and age [11, 12], Endplate changes may be a characteristic or result of the disc degeneration process, and vice versa, these changes can affect the nutrient supply to the disc and further the process of degeneration [13]. This could explain why patients in the endplate protrusion group have a higher average age than those in the non-endplate protrusion group. Furthermore, Modic type 2 changes, in particular, have a strong association with disc degeneration. This may be because this type of change reflects a longer-term and chronic process related to alterations in fat metabolism [14].

Our research shows that patients with intervertebral disc protrusion accompanied by cartilage herniation of cartilaginous endplates exhibit significant differences in back VAS scores at early postoperative stages (1 day and 1 month) and in leg VAS scores at 1 day postoperatively compared to the group without cartilage herniation of cartilaginous endplates (*P* < 0.05). However, there were no statistically significant differences in back VAS scores at 1 year postoperatively and in leg VAS scores at 1 month and 1 year postoperatively (*P* > 0.05). Previous studies have suggested that cartilage herniation of cartilaginous endplates is closely related to Modic changes, and the presence of the cartilage endplate seems to affect the absorption of the protruding intervertebral disc. In their research, cases with the presence of cartilage pieces showed less neovascularization of inflammatory granulation tissue and macrophage infiltration, which could lead to the failure of natural remission of clinical symptoms [15]. This explains why patients with cartilage herniation of cartilaginous endplates have higher VAS scores in the early postoperative period, as the inflammatory response may be more significant during this period.

Feng et al. noted that cartilage endplate tears are associated with adjacent Modic changes and endplate defects, and patients with cartilage endplate tears are more likely to experience residual back and leg pain during a 2-year postoperative follow-up. Histological characteristics reveal that the torn cartilage endplates present multiple defects, significant inflammation, and nucleus pulposus invasion, along with the upregulation of IL-1β, caspase-1, and the NLRP3 inflammasome [16]. These factors may be related to the significant differences in early postoperative VAS scores, but these differences gradually decrease over time as the inflammatory response subsides. In the early postoperative period, the inflammatory response may exacerbate pain, especially in patients with cartilage endplate damage [17]. However, over time, this inflammatory response may gradually alleviate, leading to an improvement in pain scores. This explains why, at 1 year postoperatively, there was no statistically significant difference in VAS scores between patients in the herniation of cartilaginous endplates group and those in the non-herniation of cartilaginous endplates group.

Postoperative back pain VAS scores showed significant differences between the two groups on the 1st day and 1st month after surgery (*P* < 0.05), while differences in postoperative leg pain VAS scores were statistically significant only on the 1st day, with no significant differences at 1 month or 1 year (*P* > 0.05). Kawaguchi et al. [18] investigated the characteristics of newly developed Modic changes after lumbar disc herniation surgery and their impact on early postoperative residual lower back pain. It was found that at 6 months, 28% of patients with cartilage protrusion developed new Modic changes. These patients had higher back pain VAS scores within 6 months post-surgery, but no significant difference was observed at the one-year follow-up regarding the presence or absence of Modic changes, suggesting that Modic changes may be related to early postoperative lower back pain, but this impact may decrease over time. In our Table 3, patients with Modic changes had higher postoperative back pain VAS scores compared to those without Modic changes, and the difference in back pain VAS scores at 1 month postoperatively between the two groups was statistically significant. The research on these aspects is still controversial, with some studies suggesting no significant difference in postoperative pain or functional outcomes between patients with and without Modic changes [19, 20]. In our study, six lumbar MRI features were used as criteria for diagnosing herniation of cartilaginous endplates, including Modic changes at the endplate and posterior corners, which were part of the diagnostic criteria. We believe that patients with herniation of cartilaginous endplates accompanied by Modic changes experience a higher degree of postoperative lower back pain compared to those without herniation of cartilaginous endplates, explaining why patients in the herniation of cartilaginous endplates group suffer from longer-term lower back pain after surgery.

Furthermore, We divided patients with endplate protrusions into two groups based on the presence or absence of Modic changes. The Visual Analogue Scale (VAS) is of utmost importance for assessing patients' pain on the first day after surgery. However, we observed no significant difference in the VAS scores between the two groups on the first day after surgery (*P* > 0.05). We believe that the pain variations caused by the presence or absence of endplate protrusions might have diminished the differences in pain caused by Modic changes, leading to no significant difference in VAS scores between the groups. Nevertheless, the significant differences in the Oswestry Disability Index (ODI) (*P* < 0.05) scores between the two groups indicate that the presence of Modic changes does impact the immediate functional recovery of patients post-surgery. A prospective MRI study explored the significance of Modic changes, osseous endplate injuries, and intervertebral disc degeneration as predictors of chronic lower back pain (LBP). The study found that over a 1-year follow-up period, pain decreased in most patients but increased or persisted in 36% of patients. Changes in Modic types 1 and 2 and osseous endplate injuries were associated with changes in pain intensity, while changes in Modic type 1 and osseous endplate injuries were associated with changes in ODI [21], consistent with our observations.

Some studies [22] suggest that for cases with endplate damage, PRAF, and failure at the endplate junction, the endpoint of decompression changes. Among the surgical patients participating in this study, the endpoint of surgical decompression was consistent. During the 1-year follow-up period after surgery, Most patients experienced reduced pain, but 36% of the patients had increased or persistent pain. These patients required a more aggressive surgical decompression endpoint to fully remove the endplate damage. With continuous improvements in endoscopic equipment, optical systems, surgical tools, and safety, along with enhanced diagnostic and classification methods for different spinal pathologies, Full Endoscopic Spine Surgery (FESS) techniques [23] have been able to be applied to more complex spinal lesion treatments. For patients with endplate herniation, choosing FESS might provide a better surgical view and postoperative recovery outcome.

With the enhanced recovery and aggressive surgical outcome assured, we need to identify factors which mass the initial recovery pattern. Rajasekaran et al. [24] discovered that Lumbar Disc Herniation (LDH) is more often caused by endplate junction failure (EPJF) than by annulus fibrosus rupture. The effectiveness of surgical treatments for EPJF remains an area for further research. Calcified intervertebral discs significantly [25] influence the choice of surgical method and the outcome of post-operative recovery. For Calcified Lumbar Disc Herniation (CLDH) leading to Calcified Ventral Stenosis (CVS), Posterolateral Endoscopic Lumbar Discectomy (PELD) has been proven to be a comprehensive, safe, and effective surgery.

This study faced several notable limitations, chiefly its retrospective design, which could potentially introduce biases in the selection of patients and the collection of data. As it was conducted at a single center, the generalizability of its findings to broader populations or different surgical practices remains uncertain. Additionally, the study was constrained by both its sample size and the duration of follow-up, which may undermine the strength and longevity of the conclusions reached. The lack of a control group for comparison with alternative surgical approaches or conservative management further limits the thoroughness of the results. Moreover, the primary reliance on MRI findings for categorizing patients may not fully capture the clinical subtleties associated with Lumbar Disc Herniation cases. Future studies could overcome these limitations by adopting a prospective, multicenter design with larger patient groups, longer follow-up periods, and the inclusion of control groups undergoing various treatments. Relying solely on the Oswestry Disability Index (ODI) to assess the recovery of patients' postoperative activities is limited. Other evaluation methods such as the Prolo rating for lumbar disc herniation efficacy and the Japanese Orthopaedic Association scoring system should also be adopted. Regarding the issue of whether patients undergoing surgery for cartilage endplate protrusion face an increased risk of postoperative recurrence, this is a key factor in preoperative planning and postoperative management. However, the potential impact of cartilage endplate herniation on the risk of postoperative recurrence was not addressed in this study. This issue could be a focus of future research. This would pave the way for a more definitive and broadly applicable understanding of the effects of herniation of cartilaginous endplates and Modic changes on postoperative outcomes.

## Conclusion

This study demonstrates that although patients with herniation of cartilaginous endplates may face higher pain scores in the early postoperative period, in the long term, discectomy is effective in improving the pain and functional status of all patients. Furthermore, Modic changes are closely related to the differences in postoperative pain and functional recovery among patients, suggesting that this imaging feature should be given clinical importance to more accurately predict postoperative recovery and to develop personalized treatment plans.

## Data Availability

The datasets generated and/or analysed during the current study are not publicly available due The need for follow-up studies as well as patient privacy but are available from the corresponding author on reasonable request.
